# Progress of Cholera

**Published:** 1849-01

**Authors:** 


PROGRESS OF CHOLERA.

In Berlin cholera is said to have spread less this time than it did in other epidemics, but the mortality from it has been as great. From the 27th of July to the 11th of October, the number of cases was 2,009, and of these 1262, or more than one half, were fatal.

The epidemic has broken out at Amsterdam. Seven cases had been reported at the general hospital; and four persons had died.

At Hamburgh, the official reports state that up to the 9th of October the number of persons attacked was 2,229, of whom 1,043 had fallen victims. The disease was spreading at Lubeck.

In Turkey the epidemic was gaining ground when the last accounts were received at London. It raged with great violence at Smyrna, where the mortality daily exceeded a hundred. Greece had escaped up to the last dates. In Syria and Palestine the disease was raging with great violence. At Damascus, the deaths are reported as varying from 500 to 600 daily, but the epidemic was beginning to decline. At Smyrna it had disappeared. The mortality there was to the extent of nearly two-thirds of those attacked—2,194, out of 3,212.

The disease is progressing in Scotland, and the mortality attending it is as great as elsewhere. Up to the 7th November, 468 cases had been reported, and of these 243 had terminated fatally. On the 8th 27 cases were reported; recoveries 2; deaths 24.

In England the epidemic spreads slowly, and the mortality is not alarming. The number of cases reported as having occurred in London and its vicinity on the 8th of November, was 11, of which only three were fatal.

At New York alarm was excited by the appearance of the disease a short time since among some emigrants just arrived at Staten Island. It has not been communicated, however, and the latest papers from the city are silent on the subject.

